# Global Impact of Ergot Alkaloids

**DOI:** 10.3390/toxins14030186

**Published:** 2022-03-03

**Authors:** James L. Klotz

**Affiliations:** United States Department of Agriculture-Agricultural Research Service, Forage-Animal Production Research Unit, Lexington, KY 40546, USA; james.klotz@usda.gov; Tel.: +1-859-257-1647; Fax: +1-859-257-3334

For many years, ergot alkaloids have been considered both a problem to be mitigated and a potential medical cure. These compounds have been primarily studied in the medical/pharmaceutical [1] and agricultural fields [2]. Depending on one’s perspective, the impact that ergot alkaloids have had on the progress of human medicine and livestock production can be either positive or negative. The dose or concentration of ergot alkaloid exposure is paramount. This can determine whether these compounds are implicated in the morbidity and mortality of individuals with St. Anthony’s Fire, or whether they can be used to treat migraines and post-partum bleeding; it can determine whether they are used to maximize plant resistance and persistence, or whether they constitute an animal welfare concern for grazing livestock [3,4]. The ethics of ergot alkaloid use is debated to this day, but there is no debating the impact of these compounds. Many of the positive and negative issues associated with ergot alkaloids have specific conditions with regional implications, but that does not diminish the magnitude of impact that these compounds have had on humans, livestock, and plants globally.

Research evaluating ergot alkaloids can be both basic and applied. Many types of research perspectives are necessary in understanding ergot alkaloids’ functions and how these findings might be applied. The focus of this Special Issue concerns original research and review articles that highlight benefits and detriments, and successes and failures involving ergot alkloids around the world with deference to regional distinctions. Research models range from fungus, to plant, to mammal; and the ergot alkaloids produced by both *Claviceps* and *Epichloë* spp. of fungi are included in this Special Issue. All submissions focus on ergot alkaloids’ effects (positive or negative) in different contexts. There is a benefit to this shared interest, even if the issues with ergot alkaloids do not directly overlap.

Significant advancements in the manipulation of plant–endophyte symbioses have been made in recent years to optimize the profile and concentration of the secondary compounds produced [5]. Eady [6] has reviewed the complexities plant breeders encounter when selecting a desired plant–endophyte symbiont, with New Zealand ryegrass as a model. This is a balance between selecting a source of ergot alkaloids that permit greater plant persistence, and inhibiting ergot alkaloid production that results in mycotoxicosis in grazing livestock in combination with desired plant traits. This makes the understanding of ergot alkaloid production paramount. The potential of using various-omics technologies to study ergot alkaloid production has been demonstrated in this Special Issue. In addition to traditional selection processes, Florea et al. [7] demonstrate the use of CRISPR technology to create a non-transgenic strain of *Epichloë* fungus without the genes necessary to produce ergot alkaloids. Fungi that produce ergot alkaloids can be endophytic and parasitic. Ergot contamination of cereal crops in Canadian provinces has become an issue of increasing concern. Hicks et al. [8] evaluate diversity in genes related to ergot alkaloid production in Canadian strains of the parasitic *Claviceps purpurea* to better characterize and understand the variation of ergot alkaloid content. Also looking at Canadian strains of *C. purpurea*, Liu et al. [9] evaluated the evolution patterns of gene clusters associated with different classes of ergot alkaloid production. Work of this caliber is critical to better understand this evolving issue.

Historically, human interactions with ergot alkaloids have been defined by large-scale poisonings through the consumption of contaminated grains [1]. Incidents of human ergot poisonings are increasingly rare due to improvements in crop management, grain screening and cleaning [10], and the regulation of safe quantities in food and feed [11]. However, there are still areas in the world where this can be an issue [12], and there is also still interest in the pharmaceutical potential of ergot alkaloids. Their most prominent use has been the treatment of migraines and controlling post-partum bleeding in the 18th and 19th centuries. In a current review of the past gynecological and obstetric uses of ergot alkaloids, Smakosz et al. [13] defined a potential role for the application of ergot alkaloids in modern obstetrics. In addition to clinical uses of ergot alkaloids, research assessing the sustainable production of ergot alkaloids in desirable formulations is needed. Shahid et al. [14] have developed a response surface methodology to select strains of *Penicillium citrinum* for their ability to produce ergot alkaloids in culture. Many researchers that study ergot alkaloids can relate to the challenges associated with obtaining purified forms of desired ergot alkaloids in any quantity.

Although medical applications focus on ergot alkaloids’ positive effects in humans, animal agriculture has historically and consistently viewed ergot alkaloids as a problem to be solved. Further, changing environmental conditions cause the ever-changing fungal production of ergot alkaloid profiles and concentrations. This necessitates routine surveys of grains and grasses. In this Special Issue, these are exemplified by the on-farm monitoring of ergot alkaloid levels in Kentucky horse pastures described by Lea and Smith [15], as well as the ergot alkaloids found in Slovenian feed grains, as described by Babic et al. [16]. Research of this nature is ongoing globally and contributes greatly to the mitigation of large-scale problems as well as the identification of future areas in need of research.

The variation of the content and concentration of ergot alkaloids is further complicated by livestock exposed to ergot alkaloids that demonstrate varied responses to the toxins. Poole et al. [17] and Wilbanks et al. [18] have studied various aspects, including genetics, that may make cattle more resistant to consumed ergot alkaloids. Ault-Seay et al. [19] used advanced-omics technologies to look at the rumen microbial and host metabolomes to provide a whole-animal characterization of impacts of ergot alkaloids. Mote and Filipov [20] reviewed the use of interactomics to provide a systemic understanding of the pathologies caused by ergot alkaloids that cause fescue toxicosis. A very specific pathology associated with ergot alkaloids and ergotism is a chronic vasoconstriction. Yonpaim et al. [21] looked at the acute exposure of ergot alkaloids on vasoactivity in ovine vasculature, and Valente et al. [22] evaluated prolonged ergot alkaloid exposure on the vasoactivity of bovine vasculature. Both studies [21,22] respectively evaluated aspects related to the ability of ergot alkaloids to interact with adrenergic and serotonergic receptors [23], and both papers concluded that receptor-mediated treatments for ergot alkaloid-induced vasoconstriction could be explored as potential therapies. From a systemic evaluation of ergot alkaloids’ impact on the whole animal or microbiome, to the study of a specific symptom, there is much yet to be learned about how ergot alkaloids disrupt mammalian physiology.

The collection of papers in the Global Impact of Ergot Alkaloids (https://www.mdpi.com/journal/toxins/special_issues/ergot_alkaloid) (accessed on 17 February 2022) Special Issue highlights the rich diversity of research and the complexity of the problems centered around ergot alkaloids. Although many specific issues related to accidental or intentional consumption of ergot alkaloids can be localized to a certain geographic region, the problems, challenges, and fascination with ergot alkaloids is global.
